# Cerebral microbleeds in idiopathic normal pressure hydrocephalus

**DOI:** 10.1186/2045-8118-12-S1-O5

**Published:** 2015-09-18

**Authors:** Elias Johansson, Khalid Ambaraki, Richard Birgander, Nazila Bahrami, Anders Eklund, Jan Malm

**Affiliations:** 1 Department of Pharmacology and Clinical Neuroscience, Umeå University, Sweden; 2 Department of Radiation Sciences, Umeå University, Sweden; 3 Centre for Biomedical Engineering and Physics, Umeå University, Sweden

## Introduction

Cerebral microbleeds (CMB) have been associated with dementia and small vessel disease, which also are features in idiopathic normal pressure hydrocephalus (INPH). This study aims to analyze if CMB are associated with INPH.

## Methods

Case-control study. We included 14 patients with INPH (mean age 76 years, 60% female) and 41 healthy controls (HeCo; mean age 71 years, 60% female). All were investigated with magnetic resonance imaging using a T2*-sequence. After investigation, INPH patients underwent shunt surgery. We compared the presence of ≥2 CMB between the cases and controls.

## Results

≥2 CMB were detected more frequently in the INPH group compared to HeCo (n=6, 43% versus n=4, 10%; p=0.01). Among the participants with CMB, the number of CMB was higher among the INPH patients than the HeCo (median 8; IQR 2-34 versus median 1; IQR 1-2; p=0.005). Two cases died within 30 days post-operatively; these had the highest number of microbleeds in the cohort (34 and 174 CMB).

## Conclusion

The prevalence of CMB seems to be increased in patients with INPH. The results may support a vascular component as a part of the INPH pathophysiology. The possible association between CMB and poor outcome warrants further study.

